# Early Operative Risk Assessment Using the Modified Mannheim Peritonitis Index in Patients With Generalised Peritonitis at a Tertiary Hospital in Abuja, Nigeria

**DOI:** 10.7759/cureus.69709

**Published:** 2024-09-19

**Authors:** Oladele O Situ, Onyedika Okoye, Oluwole Olaomi, D-i Akhigbe, Cephas Batta, Oluwatosin Ajibola

**Affiliations:** 1 Department of Surgery, National Hospital Abuja, Abuja, NGA; 2 Department of Emergency Medicine, Medway Maritime Hospital, Gillingham, GBR

**Keywords:** acute generalised peritonitis, emergency laparotomy, mannheim peritonitis index, modification, morbidity and mortality, nigeria

## Abstract

Introduction

The use of validated prognostic scoring tools in resource-poor environments has been hampered by the cost of procuring and maintaining devices, such as arterial blood gas analysers, to measure critical physiological derangements. Attempts need to be made to adapt these applicable risk stratification tools so they can be easily adopted in developing countries like Nigeria.

Aim

This study assessed the usefulness of a modified Mannheim peritonitis index (mMPI) as a risk stratification tool in predicting surgical outcomes in managing patients with generalised peritonitis in Abuja, Nigeria.

Methods

This was a prospective study of consecutive adult patients managed for generalised peritonitis at a tertiary hospital in Abuja over 12 months. Approval numbers NHA/EC/037/2019 and AF/013/17/110/1514 were ascribed to the project by the Institute Review Board Committee (IBR) of the National Hospital Abuja (NHA) and the National Postgraduate Medical College of Nigeria. The MPI was modified using respiratory rate and suboptimal percentage oxygen saturation (SpO_2_). Patient characteristics and treatment outcomes obtained were entered into a structured proforma, checked, and analysed using IBM SPSS Statistics for Windows, Version 25.0 (Released 2017; IBM Corp., Armonk, New York, United States). The threshold score of the modified MPI and the accuracy, sensitivity, and specificity were derived from the receiver operating characteristic (ROC) curve analysis and its coordinates. The level of statistical significance was set at a p-value of <0.05.

Results

There were 49 patients with generalised peritonitis during the study period with a male-to-female ratio of 2.5:1. The commonest cause of peritonitis in this study was penetrating abdominal injury (n=15, 30.6%), followed by complicated appendicitis (n=12, 24.5%). This study's mortality and morbidity rates were 14.3% and 63.3%, respectively. On the ROC curve, the modified MPI best predicts mortality at a threshold score point of ≥26 (accuracy of 79.4%, sensitivity of 85.7%, specificity of 61.9%, p=0.013) and morbidity at a threshold score of ≥23 (accuracy of 78.4%, sensitivity of 77.4%, specificity of 72.2%, p=0.001).

Conclusion

The modified MPI can be used as a risk assessment tool to predict postoperative outcomes in adult patients operated on for generalised peritonitis within 30 days of operative intervention at Abuja. This modification may be helpful in low-resource centres with limited access to arterial blood gas analysers. However, the original MPI might be a more accurate tool.

## Introduction

Several risk assessment tools have been validated and shown to correlate with treatment outcomes following surgery for intra-abdominal infections. They help to prognosticate and compare care outcomes, serving as critical quality improvement measures among care providers [1]. However, adopting these tools has been very limited in resource-constrained environments like Nigeria due to the high cost of the equipment necessary to assess physiological parameters that may predict the outcome of care [2,3].

One such tool is the Mannheim peritonitis index (MPI), designed by Wacha et al. in 1987 [4] and popularised by Billing et al. in 1994 [5]. This tool was specifically designed for surgical peritonitis, with a maximum achievable score of 47. Thus, patients were classified into three main severity groups, using MPI cut-off points of <21, 21-29, and >29 to correspond to mild, moderate, and severe peritonitis, respectively. According to the literature, the threshold MPI score of patients with surgical peritonitis above which there is a significant risk of fatal outcome after surgery ranges from 25 through 29 [6-8]. However, Billing et al.'s large European meta-analysis study reliably put it at a threshold score of 26 [5].

One limitation in adopting the MPI in resource-poor communities like Nigeria is the need for an arterial blood gas (ABG) analyser to determine the partial pressures of oxygen (PaO_2_) due to high procurement costs [3]. The unit price of an ABG analyser machine can be as much as $20,000 USD, excluding its unit cartridge cost and its maintenance cost [9]. This brings its use out of reach for most Nigerian hospitals, where the national health budget is 4.38% and the actual health cost per capita is $17.8 USD [10,11]. Most citizens heavily rely on out-of-pocket payments for health care. Thus, not surprisingly, the only authors that attempted to validate MPI in Nigeria excluded the use of ABG [12].

Nevertheless, the contribution of a failing lung to perioperative mortality and morbidity cannot be overlooked, as done by Nwigwe and Atoyebi in Lagos [12]. The lung is a critical organ responsible for blood oxygenation, which is crucial for the optimal functioning of all organs in the body. Therefore, its contribution to the overall outcome of patients following surgery for peritonitis must be considered. Rising respiratory rate (RR) and suboptimal percentage oxygen saturation (SpO_2_) have been described as parameters of respiratory failure that correlate with adverse outcomes. The current standard of defining lung failure is a PaO_2_ <60 mmHg or PaCO_2_ ≥50 mmHg [13]. Still, the PaO_2_/FiO_2_ ratio (especially when less than 300) has been shown to correlate with SpO_2_/FiO_2_, adjusting for the non-linear relationship between SpO_2_ and PaO_2_ (FiO2 means fractional-inspired oxygen (i.e., the proportion of the air inspired that is oxygen), while PaO2 refers to a measure of the dissolved oxygen in the blood) [14]. Furthermore, clinicians still fail to detect acute lung injury in up to 40% of critically ill patients, even with the use of ABG [15].

These observations, coupled with the cost of ABG analysis and the invasiveness of the test, which affect compliance with the use of ABG, have made us set out to determine the value of a modified MPI (using RR and SpO_2_ as early makers of lung failure) as a perioperative risk assessment tool in patients with generalised peritonitis at the National Hospital Abuja (NHA) since little is still known in this area of study in Africa.

This article was previously presented as a meeting abstract at the Association of Surgeons of Great Britain and Ireland (ASGBI) conference in Belfast on May 10, 2024 [16].

## Materials and methods

This study was a prospective single-centre cohort study of all consecutive patients with generalised peritonitis who met the inclusion criteria and were operated on at the NHA, Nigeria. Patient recruitment and data collection took place from February 2021 through January 2022. Participants were followed up for 30 days post-surgery. Study approval was received from both the Institute Review Board Committee (IBR) of the NHA (approval number: NHA/EC/037/2019) and the National Postgraduate Medical College of Nigeria (approval number: AF/013/17/110/1514). Informed consent was obtained from each study participant before enrollment. Study subjects' willingness to participate or otherwise did not affect their treatment, and all identifiable patient data were excluded from the study to maintain their anonymity. Patients' rights to withdraw were kept at every study stage, even after consent.

This study aimed to assess whether the modified MPI (mMPI) can be used as a risk assessment tool to predict postoperative morbidity and mortality among adult patients operated on for generalised peritonitis within 30 days of operative intervention. The primary outcome is mortality, and the secondary outcome is morbidity.

Inclusion criteria were patients aged ≥18 years and those with secondary peritonitis. Excluded from the study were patients younger than 18 years, patients with primary peritonitis, patients lost to follow-up, patients with no clinical evidence of suppurative peritonitis at operation, and patients discharged against medical advice within the study period.

The SpO_2_ and RR at admission on room air were used to calculate the mMPI score. Patients' ABGs were also analysed using the i-STAT® system (Abbott Point of Care Inc., Princeton, New Jersey, United States). This is to see how RR and SpO_2_ contribute to mortality compared to PaO_2_. The CG8+ Abbott i-STAT cartridges were used. Actual lung failure was defined as PaO2 <60 mmHg or PaCO2 ≥50 mmHg using ABG. To calculate the mMPI in this study, lung failure was assumed at RR of ≥30 cycles per minute or SPO2 ≤90 mmHg on room air.

All patients had laparotomy under general anaesthesia with muscle relaxation. According to the hospital protocol, ceftriaxone and metronidazole were the empiric choice of antibiotics used in this study. All blood samples for investigations were taken within the first hour of admission, and these were used to calculate the mMPI scores. The peritoneal cavity was lavaged with normal saline intraoperatively till clear.

Results were analysed using IBM SPSS Statistics for Windows, Version 25.0 (Released 2017; IBM Corp., Armonk, New York, United States). The receiver operating characteristic (ROC) curve was obtained to determine the threshold mMPI for morbidity and mortality with their corresponding accuracies. The level of statistical significance was taken as p<0.05. The resultant area under the curve (AUC), expressed in decimals, represents the percentage accuracy of the threshold score of the mMPI selected. The sensitivity and specificity of the threshold mMPI score were determined from the ROC coordinates. The 30-day primary outcome of interest was mortality, and the secondary outcome was morbidity.

## Results

A total of 3753 patients were admitted to the NHA surgical unit during the study period. Of these, only 732 had surgical operations. Of these, 49 patients underwent laparotomy for generalised peritonitis with a male-to-female ratio of 2.5:1. Table 1 below summarises the demography of the study population at the hospital presentation. 

**Table 1:** Demographic summary of the study population at hospital presentation SPO_2_: suboptimal percentage oxygen saturation; PaO_2_: partial pressures of oxygen

Variables	Mean±SD	Median	Range
Age (years)	32.9±14.8	32.0	18.0-60.0
Systolic blood pressure (mmHg)	125.1±21.2	124.0	83.0-180.0
Respiratory rate (cycles/min)	28.6±7.7	28.0	16.0-66.0
Temperature (°C)	37.6±1.0	37.5	35.6-39.3
SPO_2_ (%) in room air	95.7±3.2	96.0	88.0-100.0
PaO_2_ (mmHg)	103.9±40.0	89.0	52.0-238.0
Urine output (ml/hr)	43.3±21.4	40.0	5.0-140.0
Glasgow Coma score	14.6±2.1	15.0	4.0-15.0

The commonest cause of peritonitis in this study was penetrating abdominal injury (n=15, 30.6%), followed by complicated appendicitis (n=12, 24.5%). Other causes of peritonitis are as shown in Table 2 below. By the 30th postoperative day, seven fatalities were recorded, and 31 patients had at least one type of postoperative complication, giving this study a mortality rate of 14.3% and an overall morbidity rate of (63.3%). Table 3 shows the distribution of the morbidities observed in this study. Some patients had more than one postoperative complication. 

**Table 2:** Causes of diffuse peritonitis (postoperative diagnosis) at the National Hospital Abuja Others*: perforated gangrenous uterus post-traumatic abortion, anastomotic leak post-surgery for obstructing sigmoid tumour, anastomotic leak post-repair of typhoid ileal perforation, and blunt abdominal injury causing colonic perforation from a road traffic accident PUD: peptic ulcer disease

Postoperative (final) diagnosis	Frequency (n=49)	Percent
Penetrating abdominal injuries	15	30.6
Ruptured appendix	12	24.5
PUD perforation	10	20.4
Perforated typhoid ileitis	6	12.2
Gangrenous ileal volvulus from adhesions	2	4.1
Others*	4	8.2

**Table 3:** Distribution according to morbidities Others*: acute kidney injury (two patients), bladder injury, COVID-19, on-table cardiac arrest, short bowel syndrome (SBS), acute respiratory distress syndrome (three patients), and septic shock

Complications	Frequency (n=80)	Percent
Surgical site infection (superficial, deep, and organ space)	22	27.5
Prolonged admission (≥14 days)	20	25
Malnutrition	11	13.8
Wound breakdown	9	11.2
Pneumonia	5	6.3
Prolonged ileus (>5 days)	2	2.5
Anastomotic leak	1	1.3
Others*	10	12.5

mMPI scores of patients recruited ranged from 10 to 37 with an average of 24.84±6.21. In general, patients who survived had a significantly lower mean mMPI score of 23.4±5.9 compared to non-survivors with a mean mMPI score of 30.1±4.5 (t=3.218, p=0.009). Mortality rates were 30% in the mMPI score group >29, 14.8% in the mMPI score group 21-29, and 0% in the mMPI score group <21.

Factors associated with mortality in this study include admitting Glasgow Coma score <15, urine output <25 ml/hr, PaO_2_ <60 mmHg, prolonged admission of ≥14 days, and patients requiring ICU admission, as described in Table 4.

**Table 4:** mMPI risk factors associated with mortality f*: Fisher's exact test; ꭓ^2^: chi-squared test; PaO_2_: partial pressures of oxygen; mMPI: modified Mannheim peritonitis index

Variable	ꭓ^2^	P-value
PaO_2_ <60 mmHg (yes or no)	13.112	0.007^f*^
Urine output (<25 or ≥25 ml/hour)	9.503	0.017^f*^
ICU admission (yes or no)	15.320	0.002^f*^
Prolonged admission (<14 days or ≥14 days)	6.815	0.014^f*^
Glasgow Coma score (<15 at admission or 15)	12.511	0.018^f*^

Notably, RR ≥30 cycles/min (p=0.685), percentage SpO_2_ ≤90% (p=0.554), nature of peritoneal exudate (p=0.136), malnutrition (p=0.178), malignancy (p=143), gender (p=0.656), age ≥50 years (p=0.554), and organ failure (p=0.417) were all not associated with mortality. Spearman's correlation analysis showed a positive relationship between the mMPI score and mortality (r=0.358, p=0.012), presence of morbidity (r=0.476, p=0.001), number of complications (r=0.550, p=0.000), length of ICU stay (r=0.376, p=0.008), and length of hospital stay (r=0.469, p=0.001). Table 5 below shows that higher mMPI score groups were significantly associated with the occurrence of morbidity within the 30-day postoperative period, the number of complications/morbidities, and prolonged hospital admission.

**Table 5:** Association between outcome of surgery and mMPI score *: significant at 95% (p<0.05; Fisher's exact test was used); χ^2^: chi-squared test; mMPI: modified Mannheim peritonitis index

Postoperative outcomes	mMPI score group (n=49)			χ^2^	P-value
	<21 n=12 n(%)	21-29 n=27 n(%)	>29 n=10 n(%)		
Mortality					
Yes	0 (0)	4 (14.8)	3 (30)	4.023	0.134
No	12 (100)	23 (85.2)	7 (70)		
Morbidities					
Yes	6 (50)	15 (55.6)	10 (100)	7.406	0.025^*^
No	6 (50)	12 (44.4)	0 (0)		
Number of complications					
<2	10 (83.3)	18 (66.7)	2 (20)	9.966	0.007^*^
≥2	2 (16.7)	9 (33.3)	8 (80)		
Multiple surgeries					
Yes	0 (0)	4 (14.8)	2 (20)	2.400	0.301
No	12 (100)	23 (85.2)	8 (80)		
ICU stay					
No	12 (100)	24 (88.9)	7 (70)	4.640	0.098
Yes	0 (0)	3 (11.1)	3 (30)		
Hospital stay					
<14	9 (75)	18 (66.7)	2 (20)	8.224	0.016^*^
≥14	3 (25)	9 (33.3)	8 (80)		
Residual disease					
None	10 (83.3)	21 (77.8)	4 (40)	7.892	0.096
Yes	2 (16.7)	2 (7.4)	3 (30)		

In this study, 22 patients were identified as having "respiratory failure" using the mMPI risk assessment tool, but only four patients were confirmed with PaO_2_ <60 mmHg using the ABG analyser. Additionally, three patients met the acute respiratory distress syndrome criteria using the Berlin definitions (PaO_2_/FiO_2_ <300). A sub-analysis (see Table 6 below) using different RR thresholds (including ≥49 cycles/min) did not show a significant association with mortality. However, patients with admitting RR ≥30 cycles/min had significantly prolonged hospital admission ≥14 days (ꭓ^2^=8.607, p=0.004), and PaO_2_ at 70 mmHg was significantly associated with mortality (p=0.004).

**Table 6:** A sub-analysis of the association between outcome (mortality and morbidity), respiratory rate, and PaO2 among patients with generalised peritonitis at the National Hospital Abuja x^2^: chi-squared test; PaO_2_: partial pressures of oxygen in blood; f: Fisher's exact test value

Patient characteristics at admission	Mortality (n=49)		X^2^	P-value
	No	Yes		
Respiratory rate at the threshold of 35 (cycles/minute)				
<35	38 (90.5%)	7 (100%)	0.726	1.000^f^
≥35	4 (9.5%)	0 (0%)		
Respiratory rate at the threshold of 49 (cycles/minute)				
<49	41 (97.6%)	7 (100%)	0.170	1.000^f^
≥49	1 (2.4%)	0 (0%)		
PaO_2_ (mmHg)				
>70	41 (97.6%)	4 (57.1%)	13.112	0.007^f^
≤70	1 (2.4%)	3 (42.9%)		
Patient characteristics at admission	Prolonged admission		X^2^	P-value
	<14 days	≥14 days		
Respiratory rate at the threshold of 30 (cycles/minute)				
<30	21 (72.4%)	6 (30%)	8.607	0.004^f^
≥30	8 (27.6%)	14 (70%)		

**Figure 1:**
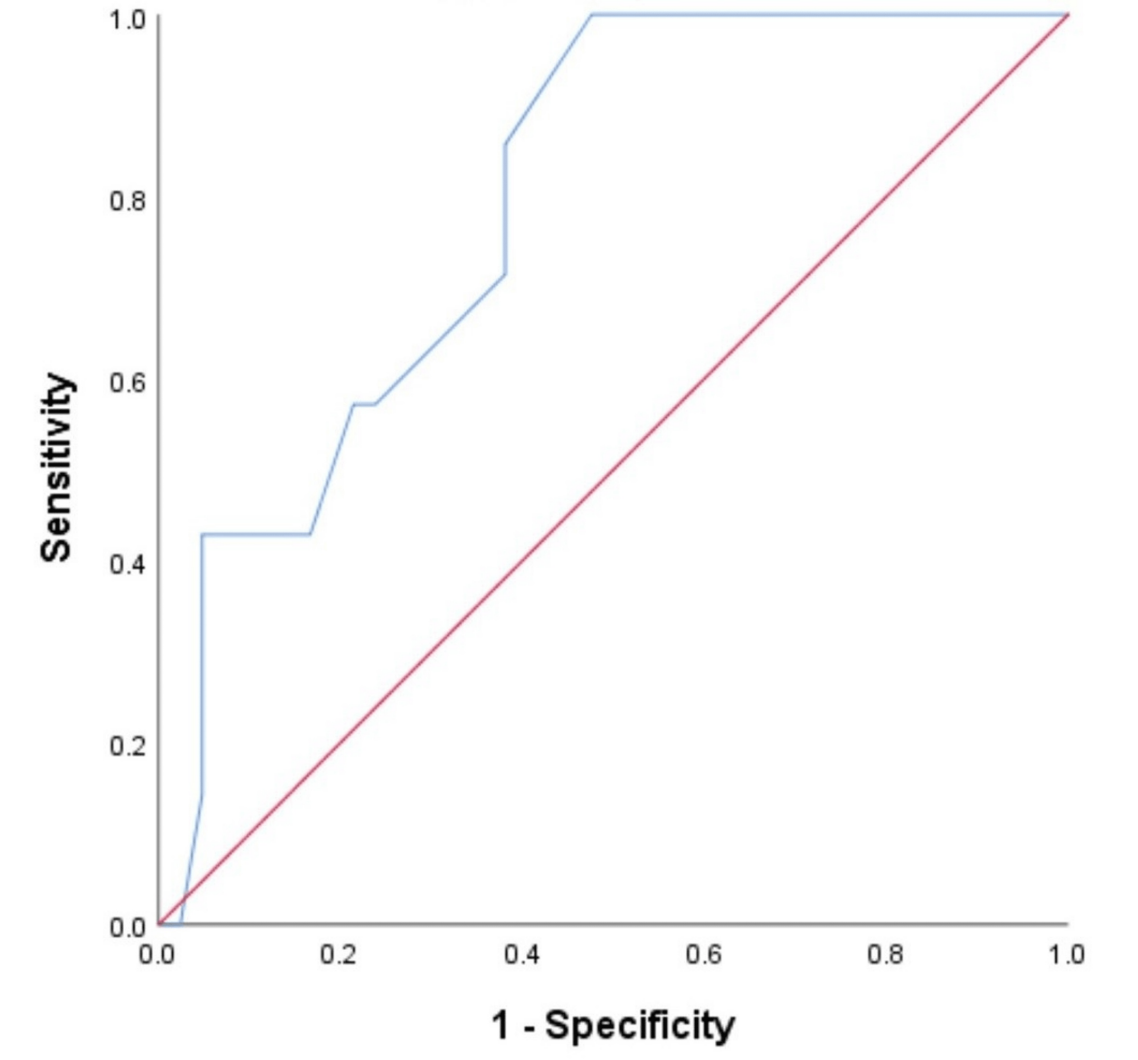
A graph showing the mMPI ROC curve for mortality (AUC=0.794, sensitivity=85.7%, specificity=61.9%, 95% confidence interval) ROC: receiver operating characteristics; AUC: area under the curve (this represents the accuracy (79.4%) of the mMPI to predict mortality at the identified threshold of 26 correctly); mMPI: modified Mannheim peritonitis index

**Figure 2:**
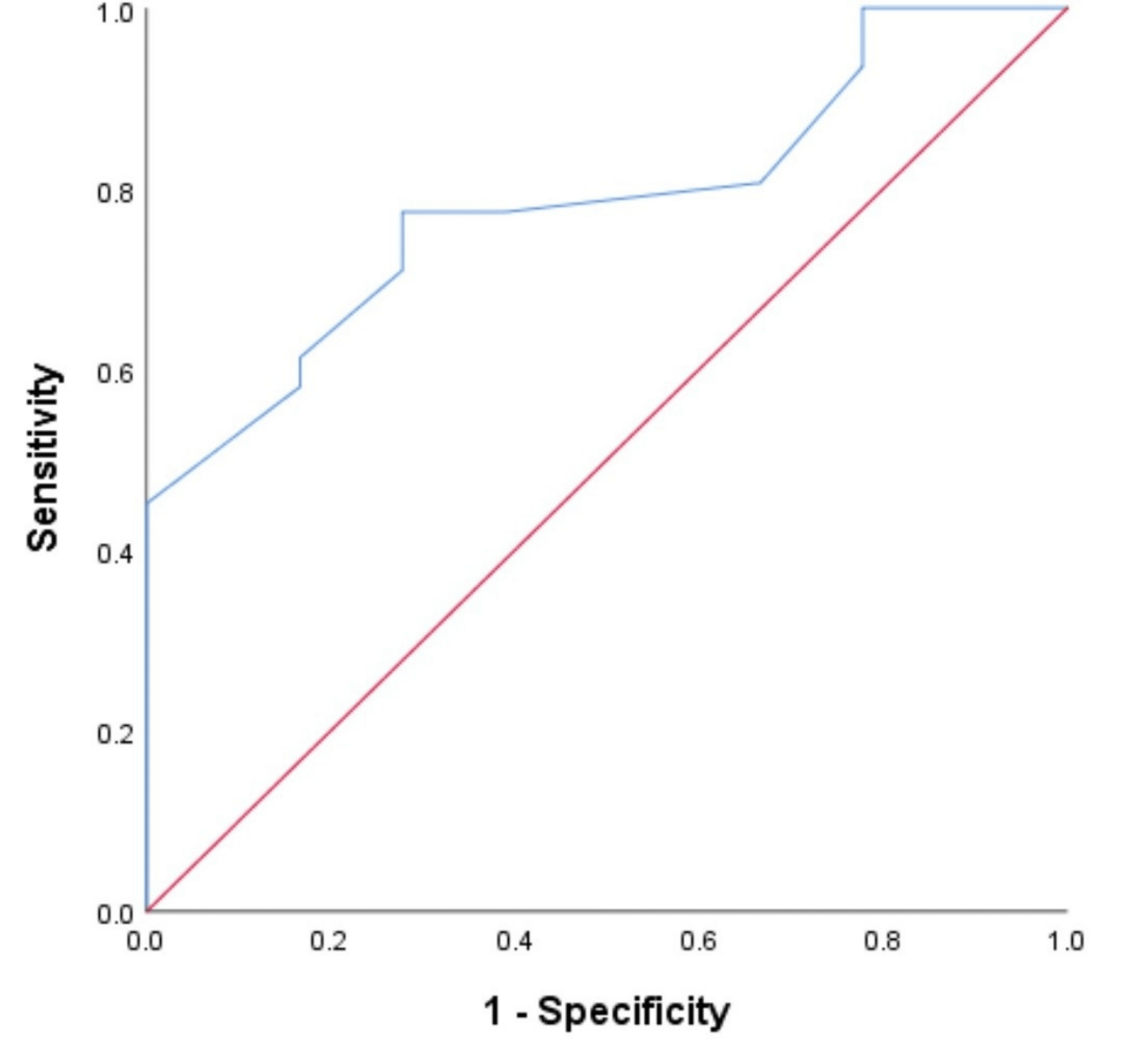
A graph showing the mMPI ROC curve for morbidity (AUC=0.784, sensitivity=77.4%, specificity=72.2%, 95% confidence interval) ROC: receiver operating characteristics; AUC: area under the curve (this represents the accuracy (78.4%) of the mMPI to predict morbidity at the identified threshold of 23 correctly); mMPI: modified Mannheim peritonitis index

The ROC curve for mortality (Figure 1) and morbidity (Figure 2) identified a threshold mMPI of 26 (p=0.013) and 23 (p=0.001) for mortality and morbidity, respectively.

## Discussion

This was a one-year prospective study carried out at the NHA to assess the usefulness of the mMPI as a risk assessment tool in predicting treatment outcomes in adult patients with generalised peritonitis. The mean mMPI score of survivors in this study was significantly lower than that seen in patients who died (p=0.009). However, unlike the use of the original MPI, this study demonstrated that the rising mMPI scores of the study participants had a stronger significant positive relationship with the occurrence and number of morbidities than it does for mortality [6,17].

The mean mMPI (24.8±6.2) score of patients in this study lies within the general quoted mean ranges for the original MPI, which lies between 17.5 and 31 [18-21]. Also, the mean mMPI for survivors (23.4±5.9) and non-survivors (30.1±4.5) in this NHA study is still within the usually reported ranges for the original MPI in many studies [19,20,22]. Howbeit, the mean mMPI score in this NHA study was higher than those described by Nwigwe and Atoyebi in Lagos because we ascribed weighted scores to the lung function in terms of RR and SpO_2_ in this study rather than deleting these parameters [12].

Contrary to the described importance of RR as a marker of lung function and mortality [23-25], overall, our modification of MPI using RR ≥30 cycles/min and SpO_2_ ≤90% seems to correlate better with the occurrence and number of morbidities rather than mortality. With our modification of MPI, Spearman's correlation analysis only showed a weak relationship between the increasing mMPI grouping and mortality (r=0.358, p=0.012), and the association between the rising mMPI score group and mortality did not reach a statistically significant level (*X*^2^=4.023, p=0.134). This observation might have resulted from overestimating the number of patients with respiratory failure using RR and SpO_2_ parameters alone, as PaO_2_ only identified four patients in this study as having respiratory failure, which was significantly associated with mortality, just as observed by Khan et al. [26].

Again, considering the RR of ≥49 cycles/min as one of the defining criteria for respiratory failure, as proposed by Knaus et al. [27], it was still not statistically associated with mortality (p=1.000) in this study, as only one patient had an RR over 49 cycles/min. It is, however, interesting knowing that even an admitting PaO_2_ of <70 mmHg was significantly associated with mortality (p=0.007), giving credence to the value of PaO_2_ as a more reliable marker of respiratory failure than RR and SpO_2_ (see Table 6). This, therefore, begs the question: "Will deleting the lung failure assessment on the original MPI be a better modification in predicting outcome than replacing it with SPO_2_ and respiratory rate?"

The demonstrated threshold mMPI point on the ROC curve for mortality and morbidity in this study is within the ranges of previous studies where the original MPI was used [5,28]. The varying threshold cut-off points expressed by different authors could reflect the different demography of the patients involved and the inclusion criteria used. Generally, the threshold mMPI score of 26 for mortality in this study agrees with the peritonitis study group's classic work, which describes the threshold of severe peritonitis with an associated higher risk of death and morbidity [5]. The AUC reflects the accuracy of the test using the ROC, and the closer the test is to 1.0 (i.e., a perfect tool), the more reliable the tool is (an AUC of 0.7-0.8 is regarded as an acceptable tool), hence, the recommendation of our mMPI with an accuracy of 79.4%, like the original MPI of Billing et al. that has an accuracy of 83% at a threshold score of 26 [5]. The ROC curve coordinates show the threshold's sensitivity and specificity, which is still acceptable in our study.

The importance of this study is that our MPI modification may predict morbidity and mortality in patients with generalised peritonitis in areas without ready access to the ABG analyser machine to calculate the original MPI score. However, this proposed mMPI at NHA may be more associated with the risk of postoperative morbidity than the risk of mortality. Patients with mMPI scores ≥26 at admission may be regarded as having severe peritonitis with an increased risk of fatality after surgery. Those patients with mMPI scores ≥23 have a higher risk of postoperative complications. Furthermore, some researchers [29] have reported mortality rates of zero with the original MPI score <21, including this NHA study. Thus, patients with mMPI or MPI score <21 may be regarded as having a low risk of mortality.

A limitation of this study is the small sample size and its single-centre study. The small sample size and, hence, power of this study might not have revealed some known risk factors associated with mortality. 

## Conclusions

The mMPI can be used as a cheap, quick, and easy-to-use risk assessment tool in predicting the outcome of treatment in adult patients operated on for generalised peritonitis at NHA within 30 days of operative intervention. mMPI scores were significantly associated with the risk of mortality at a threshold ≥26 and morbidity at a threshold score of ≥23. However, hypoxaemia (PaO_2_ <60 mmHg), as assessed in the ABG analysis, yielded a better assessment of lung failure than RR and SpO_2_ and is more associated with the risk of death. Because of this, the original MPI using the ABG may be a more accurate prognosticating tool for mortality compared with our modification.
